# Anti-emetic mechanisms of zingiber officinale against cisplatin induced emesis in the pigeon; behavioral and neurochemical correlates

**DOI:** 10.1186/s12906-015-0556-0

**Published:** 2015-02-26

**Authors:** Ihsan Ullah, Fazal Subhan, Muhammad Ayaz, Rehmat Shah, Gowhar Ali, Ikram Ul Haq, Sami Ullah

**Affiliations:** Department of Pharmacy, University of Swabi, Swabi, Pakistan; Department of Pharmacy, University of Peshawar, Peshawar, Pakistan; Department of Pharmacy, University of Malakand, Chakdara, Dir Lower Pakistan; National Institute of Health, Islamabad, Pakistan; Hospital Pharmacist, Khyber Teaching Hospital, Peshawar, Pakistan

**Keywords:** Cisplatin, Emesis, *Zingiber officinale*, Pigeon, Serotonin

## Abstract

**Background:**

*Zingiber officinale* (*ZO*, family *Zingiberaceae*) has been reported for its antiemetic activity against cancer chemotherapy induced emesis in animal models and in clinics. Current study was designed to investigate *ZO* for potential usefulness against cisplatin induced vomiting in pigeon and its effects on central and peripheral neurotransmitters involved in the act of vomiting.

**Methods:**

*Zingiber officinale* acetone fraction (*ZO*-ActFr) was investigated for attenuation of emesis induced by cisplatin in healthy pigeons. Neurotransmitters DA, 5HT and their metabolites DOPAC, HVA and 5HIAA were analyzed using High Performance Liquid Chromatography system coupled with electrochemical detector in area postrema, brain stem and intestine. Antiemetic effect of *ZO*-ActFr was correlated with central and intestinal neurotransmitters levels in pigeon.

**Results:**

Cisplatin (7 mg/kg i.v.) induced emesis without lethality upto the observation period. *ZO*-ActFr (25, 50 & 100 mg/kg) attenuated cisplatin induced emesis ~ 44.18%, 58.13% (P < 0.05) and 27.9%, respectively; the reference drug, metoclopramide (MCP; 30 mg/kg), produced ~ 48.83% reduction (P < 0.05). *ZO*-ActFr reduced (P < 0.05 - 0.001) 5-hydroxytryptamine (5HT) concentration in the area postrema, brain stem and intestine at 3^rd^ hour of cisplatin administration, while at the 18^th^ hour *ZO* treatments attenuated the dopamine upsurge (P < 0.001) caused by cisplatin in the area postrema and 5HT concentration (P < 0.01 - 0.001) in the brain stem and intestine. *ZO* treatments alone did not altered the basal neurotransmitters and their metabolites in the brain areas and intestine.

**Conclusion:**

The behavioral study verify the antiemetic profile of *ZO* against cisplatin induced emesis in the pigeon, where central and peripheral neural evidences advocate the involvement of serotonergic mechanism at initial time point (3^rd^ hr), while the later time point (18^th^ hr) is associated with serotonergic and dopaminergic component in the mediation of its antiemetic effect.

## Background

Cytotoxic agents like cisplatin and cyclophosphamide are having the side effects of nausea and vomiting most feared by patients undergoing cancer chemotherapy [1]. The D_2_ receptor blocker “metoclopramide” was found to be effective against Chemotherapy Induced Vomiting (CIV) at higher doses, where the anti-emetic effect is reported to be mediated through antagonism of 5-hydroxy tryptamine type 3 (5HT_3_) receptors [2,3]. These findings of 5HT_3_ mediated anti-emetic effect of metoclopramide led to the discovery of 5HT_3_ receptor antagonists.

The failure of single anti-emetic agent for the control of CIV is steering the etiology to be multifactorial, and there are evidences for the involvement of many neurotransmitter systems including serotonergic [4,5], dopaminergic [6,7] and neurokininergic [8,9] systems acting in emetic circuitry at different time pointes. In the past decades, major advances have been made in the understanding of the neuro-pharmacology of the emetic pathways.

The neurotransmitter “Serotonin” (5HT) is the primary culprit in the initiation of vomiting response especially considering CIV [10]. Upto 95% of 5HT is present in the enterochromaffin (EC) cells in the gastrointestinal mucosa along with substance P [11,12], which is released by the noxious stimulus caused by Highly Emetogenic Chemotherapy (HEC) agents like cyclophosphamide and cisplatin [5,13]. Furthermore, in human and animal studies, there are evidences for the increased level of 5-Hydroxy Indole Acetic Acid (5HIAA, urine) [14,15], 5HT in the intestinal mucosa (ileal segment), Tryptophan Hydroxylase (TPH, ileum), Aromatic L-amino Decarboxylase (AADC, ileum) [16] and in the brain stem following cisplatin treatment, while a decrease in Monoamine Oxidase (MAO, ileum) has also been reported [16]. This enhancement in 5HT biosynthesis and reduction in degradation ultimately lead to the upsurge of serotonin which is imperative in the mediation of vomiting act.

Dopamine (DA) is also among the several neurotransmitters, which theater its role in the genesis of vomiting, through selective activation of D_2_ receptors, localized in the limbic system, hypothalamus, amygdala and in the brain stem emetic circuitry [17,18]. Dopaminergic agonists like morphine and apomorphine has been reported to be emetic in a variety of species including dogs [19], ferrets [6,20] and human [21]. The emetic action of apomorphine and loperamide has been suggested to be mediated in the chemoreceptor trigger zone/area postrema through stimulation of dopamine receptors, as ablation of this area abolished the vomiting response [17,22].

Plants are proving themselves as important therapeutic entities that are economical, safe and readily available particularly in rural communities [23-26]. *Zingiber officinale* (Ginger) has been used as a natural herb in the treatment of vomiting for more than 2000 years in China and as common spice for cooking in Asian countries [27]. One of its therapeutic indications has always been in the treatment of nausea and vomiting, as its carminative, spasmolytic and aromatic properties suggest its direct effects on the gastrointestinal system. The major components present in ginger extract which is a mixture of homologues having 10, 20 and 14 carbon atoms in side chain that are designated as gingerols [28]. Gingerols, in particular 6-gingerol has been identified as the major active constituent responsible for its characteristic taste and is reported to enhance gastrointestinal motility and have capability to antagonize 5HT_3_ receptors in the gastrointestinal tract (GIT) [29], which supports its anti-emetic property.

Keeping in view the gastroprokinetic and 5HT_3_ receptor antagonist property, the present study was designed to screen the intrinsic anti-emetic activity of *Zingiber officinale* (*ZO*) against cisplatin induced emesis in pigeon. Furthermore, considering the relevance of serotonin and dopamine in emesis, this study was extended to evaluate the participation of these monoamine neurotransmitters and their metabolites in cisplatin induced vomiting, and to examine the impact of *Zingiber officinale* (*ZO*) on serotonin, dopamine and their metabolites centrally in specific brain areas involved in the act of vomiting and peripherally in the intestine in pigeon.

## Methods

### Animals

Pigeons of either sex (mixed breed, Department of Pharmacy, University of Peshawar, Pakistan) weighing between 250 - 350 g were used. They were housed in groups of eight at 22–26°C under a 12 h light/dark cycle and had free access to food (locally available food; Millet + Wheat) and water before and during experimentation. All the experimental procedures were approved by the Ethical Committee of the Department of Pharmacy, University of Peshawar (Ref. No 5/EC/Pharm; Dated: June 15, 2011) and are in accordance with the UK Animal Scientific Procedure Act, 1986.

### Drugs and chemicals

High Performance Liquid Chromatography (HPLC) grade acetonitrile, methanol, 1-octane sulphonic acid sodium salt (Fisher scientific U.K.), sodium dihydrogen orthophosphate and ethylene diamine tetra acetic acid (EDTA) were purchased from the Merck local distributor in Peshawar, Pakistan. Noradrenaline, DOPAC, Dopamine, 5HIAA, HVA, serotonin were from Acros organics, Belgium. Cisplatin was from Korea United Pharm. Inc. (Korea) and was dissolved in normal saline at 65 - 70°C. Metoclopramide (MCP) was purchased in solution from GlaxoSmithKline (GSK Pakistan Ltd.). Acetone was from Haq Chemicals Peshawar (Pakistan). The plant rhizome were purchased from a local market at Mardan and was authenticated by Prof. Dr. Muhammad Ibrar, Department of botany, University of Peshawar.

### Extraction of *Zingiber officinale*

**Figure 1:**
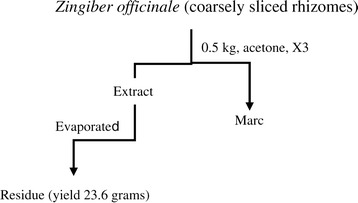
**Extraction scheme for** ***Zingiber officinale*** **acetone fraction.**

A total of 0.5 kg of fresh ginger was bought from the central vegetable market in Mardan, Pakistan. A sample was deposited at the Herbarium of the Department of Botany, The University of Peshawar, Peshawar, with the voucher number (voucher No 20017 - pup). Ginger was washed for any contaminants and then sliced to expose the inner part. It was then soaked in 2 L of acetone and kept for a total of 3 days, thrice. The combined filtrate was concentrated in a rotary evaporator to obtain a thick extract with a yield of 4.72% (Figure 1).

### Drug formulation

Cisplatin was dissolved in normal saline by heating up to 60°C, cooled upto 40 - 45°C and immediately administered. The acetone fraction of *Zingiber officinale* was dissolved in normal saline by gentle agitation and sonicated to get clear solution and administered.

### Drug administration

Intravenous and intramuscular routes were used for drug administration via brachial wing vein and chest muscle, respectively. Immediately, after the last injection, the animals were put back in the specially designed confining/observation cages and the number of emesis episodes and latency to first vomit were recorded for 24 h. ZO-ActFr and MCP or respective vehicles, were administered 30 minutes before cisplatin administration. At the end of experiment, body weight loss was calculated. Subsequently, the animals were decapitated to terminate the experiment.

### Anti-emetic assay

On the day of experiment, the pigeons were placed in individual cages specially designed for video observation. After cisplatin (7 mg/kg) [30] administration at t = 0 [31] the behavior of the pigeon was recorded for 24 h. Food and water were available during the observation period and each animal was used only once. The response with or without oral expulsion was considered as one vomiting episode [32]. A vomiting episode was considered completed when the pigeon adapted relaxed posture.

### Tissue sampling for neurotransmitters analysis

At the end of each experiment, animals were decapitated and the brain areas and intestinal samples 5 – 6 cm from the pylorus were rapidly collected and placed on a cold ice plate (0°C). The dissection of brain parts was carried out according to the atlas of Karten and Hodos [33] and Henri M. Duvernoy [34]. The collected brain tissue samples were first cleared of vessels and meninges, while fecal matter and mesenteries were carefully removed from intestinal samples, were weighed and stored at - 80°C until analysis.

### Determination of neurotransmitters and their metabolites

Tissue samples were homogenized in cold 0.2% perchloric acid (PCA) at 5000 rpm with the help of Teflon glass homogenizer (Wise stir HS 30 E). After centrifugation (Centurion UK) at 12000 g/min (4°C) and filtered through a 0.45 micron filter. Neurotransmitters and their metabolites were analyzed using High Performance Liquid Chromatography system (HPLC, Shimadzu, Japan) coupled with Electrochemical Detection (ECD, ESA Coulochem III model 5300), a pump (model LC-20AT), and an analytical column (Teknokroma 3 × 150, 3um). Electrodes 1 and 2 of the analytical cell were set at + 200 and – 200 mV respectively, with a sensitivity of 2 mA, while the guard cell (model 5020) potential was set at 500 mV. The mobile phase consisted of 94 mM sodium dihydrogen orthophosphate, 40 mM Citric acid, 2.3 mM sodium 1-octane sulphonic acid, 50 uM EDTA, and 10% acetonitrile (pH 3). The flow rate was maintained at 0.6 mL/min. The limit of detection was 11 pg for each of the neurotransmitter and their metabolites except for HVA which was 19 pg. The standards used were noradrenaline hydrochloride (NA), 3, 4-dihydroxyphenylacetic acid (DOPAC), dopamine hydrochloride (DA), 5-hydroxyindole-3-acetic acid (5HIAA), Homovanillic acid (HVA) and serotonin (5HT) [35].

### Statistical analysis

The differences between means were evaluated using a one way analysis of variance (ANOVA) followed by Dunnett or Tukey multiple comparison tests. P < 0.05 was considered as statistically significant. The animals which showed complete suppression of R + V were not included in statistical analysis for latency. Data represent the mean ± s.e.m. unless otherwise indicated.

## Results

### Effect of ZO-ActFr or MCP on cisplatin induced Retching plus Vomiting (R + V)

*Zingiber officinale* acetone fraction (ZO-ActFr) at the dose of 50 mg/kg provided maximum protection against the R + V episodes which was ~ 58.13% (18 ± 4.2 episodes) (P < 0.05) as compared to cisplatin control. The attenuation with the 25 & 100 mg doses observed was 44.18% (24 ± 4.1 episodes) and 27.9% (31 ± 5.6 episodes) respectively, but the suppression was found to be statistically non-significant (Table 1). The standard MCP reduced the R + V episodes ~ 48.83% (22 ± 4.3 episodes) (P < 0.05) and significantly increased (P < 0.01) the latency time as compared to cisplatin control.

**Table 1:** **Effect of ZO-ActFr or MCP on cisplatin induced R + V and jerking during a 24 h observation period**

**Drug treatment**	**Dose and route**	**Pigeons n/ vomited**	**R + V Mean ± sem**	**Latency (min) Mean ± sem**	**Jerks Mean ± sem**	**Wt loss (%)Mean ± sem**
Saline + Cisplatin	02 ml/kg i.m. + 07 mg/kg i.v.	6/6	43 ± 4.3	68 ± 3.7	407 ± 64	16.6 ± 1.8
MCP + Cisplatin	30 mg/kg i.m. + 07 mg/kg i.v.	8/8	22 ± 4.3*	230 ± 84**	447 ± 103	11.5 ± 1.5
ZO-ActFr + Cisplatin	25 mg/kg i.m. + 07 mg/kg i.v.	7/7	24 ± 4.1	124 ± 21	246 ± 92	8.1 ± 1.0*
	50 mg/kg i.m. + 07 mg/kg i.v.	7/7	18 ± 4.2*	77 ± 15	376 ± 97	11.3 ± 2.3
	100 mg/kg i.m. + 07 mg/kg i.v.	8/8	31 ± 5.6	85 ± 15	569 ± 125	9.1 ± 1.6

*p < 0.05, **p < 0.01 (ANOVA followed by Tukey post hoc test).

**Figure 2:**
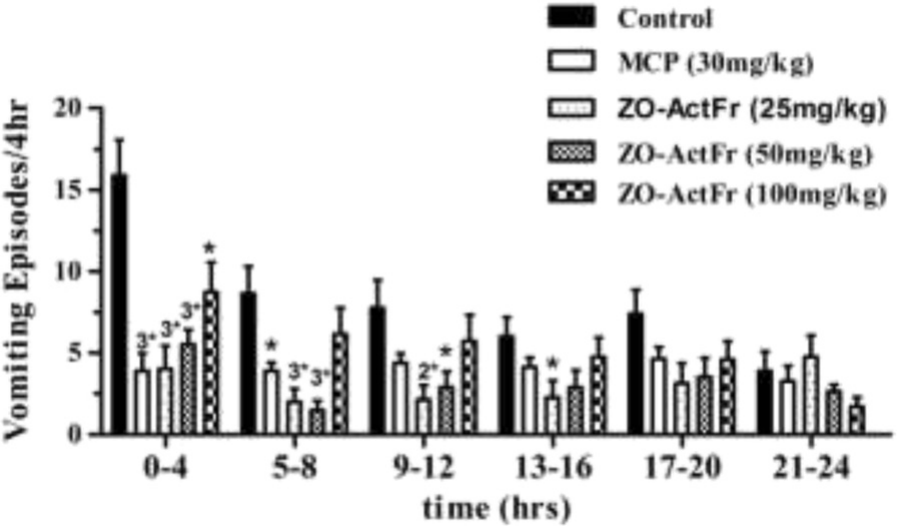
**The effect of** ***Zingiber officinale*** **acetone fraction (ZO-ActFr; 25, 50 & 100 mg/kg) and standard metoclopramide (MCP; 30 mg/kg) on cisplatin induced Retching plus Vomiting (R + V) during a 24 hr observation period.** Each bar represents the mean ± s.e.m of R + V episodes occurring during 4 hr periods (n = 6–8).

In this study the standard MCP and the treatments failed to provide complete vomiting suppression, as all the animals tested showed the vomiting response. ZO-ActFr 25 & 50 mg provided protection upto 16 hr and 12 hr, respectively, while standard MCP was also found effective upto 12 hr of observation period (Figure 2).

### Effect of ZO-ActFr or MCP on cisplatin induced jerks and weight loss

*Zingiber officinale* acetone fraction (ZO-ActFr) did not reduced jerking episodes at any dose treated and similarly the standard MCP also failed to reduce the jerking episodes as compared to cisplatin control. In cisplatin control group animals lost upto 16% of their starting body weight, while in treated groups only ZO-ActFr at the dose of 25 mg attenuated the weight loss significantly (P ˂ 0.05), while with other dose treatments the reduction observed was statistically non-significant (Table 1).

### Effect of standard MCP or ZO-ActFr on basal level of neurotransmitters and their metabolites in the brain areas and small intestine

The standard MCP treatment reduced the concentration of 5HIAA in the areas of AP and BS with significance of (P < 0.05) and (P < 0.001), respectively as compared to basal level. In addition, the decrease in the concentration of HVA was also observed in the AP (P < 0.05) with respect to basal HVA concentration (Table 2). ZO-ActFr (50 mg/kg) did not altered the basal neurotransmitter level except a decrease in the concentration of 5HIAA in the brain area of BS, where the difference was found to be statistically significant (P < 0.05) as compared to basal level (Table 2).

**Table 2:** **Effect of ZO-ActFr (50 mg/kg) administered 30 minutes before saline administration, on the basal level of neurotransmitters and their metabolites (ng/mg tissue wet weight) in the brain areas and intestine in pigeons at t = 3 hr (n = 6–8)**

**Treatment**	**NA**	**DOPAC**	**DA**	**5HIAA**	**HVA**	**5HT**
**Area postrema**						
Saline	0.610 ± 0.014	0.382 ± 0.111	0.590 ± 0.146	0.158 ± 0.036	0.913 ± 0.095	0.062 ± 0.034
MCP 30 mg	0.023 ± 0.005	0.017 ± 0.006	0.025 ± 0.012	0.005 ± 0.001*	0.121 ± 0.063*	0.023 ± 0.001
ZO-ActFr 50 mg	0.372 ± 0.036	0.122 ± 0.047	0.191 ± 0.037	0.039 ± 0.006	0.874 ± 0.204	0.045 ± 0.008
**Brain stem**						
Saline	0.094 ± 0.022	0.060 ± 0.020	0.175 ± 0.078	0.060 ± 0.021	0.060 ± 0.016	0.010 ± 0.003
MCP 30 mg	0.119 ± 0.033	0.027 ± 0.006	0.044 ± 0.012	0.007 ± 0.001***	0.066 ± 0.031	0.019 ± 0.002
ZO-ActFr 50 mg	0.056 ± 0.055	0.128 ± 0.127	0.082 ± 0.054	0.011 ± 0.001*	0.088 ± 0.021	0.020 ± 0.001
**Intestine**						
Saline	0.194 ± 0.059	0.067 ± 0.020	0.090 ± 0.064	0.076 ± 0.058	0.056 ± 0.025	0.049 ± 0.016
MCP 30 mg	0.138 ± 0.039	0.054 ± 0.025	0.059 ± 0.018	0.097 ± 0.022	0.198 ± 0.102	0.062 ± 0.013
ZO-ActFr 50 mg	0.334 ± 0.131	0.037 ± 0.028	0.356 ± 0.113	0.050 ± 0.008	0.231 ± 0.110	0.103 ± 0.015

Standard MCP is also shown.

Values significantly different compared to basal level (saline) are indicated as *p < 0.05, ***p < 0.001 (ANOVA followed by Tukey post hoc analysis).

### Effect of standard MCP or ZO-ActFr on the level of neurotransmitters and their metabolites in the brain areas and small intestine at 3^rd^ hour of cisplatin administration

Cisplatin treatment significantly increased (P < 0.001) the concentration of 5-hydroxy tryptamine (5HT) in the brain stem (BS) and intestine as compared to basal level, while a non-significant increase was observed in the area postrema (AP; Table 3). The treatment with standard MCP (30 mg/kg) failed to change the concentration of NA, DOPAC, DA, 5HIAA and HVA in all the brain areas (AP & BS) and intestine in comparison to saline control, but reduced the concentration of 5HT in the BS and intestine significantly (P < 0.001) as compared to cisplatin control (Table 3). In addition to its inhibitory effects on 5HT, MCP also decreased 5HIAA concentration in both the brain areas (AP & BS) and intestine significantly (P < 0.01-0.001, Table 3). ZO-ActFr (50 mg/kg) reduced the level of 5HIAA (P < 0.001) and 5HT (P < 0.05 - 0.001) in the brain (AP & BS) and intestine as compared to cisplatin control. No significant alteration was seen in NA, DOPAC, DA and HVA in the brain (AP & BS) and intestine (Table 3).

**Table 3:** **Effect of ZO-ActFr (50 mg/kg) or standard MCP (30 mg/kg) administered 30 mins before cisplatin challenge, on the level of neurotransmitters and their metabolites (ng/mg tissue wet weight) in the brain areas and intestine in pigeons at t = 3 hr of cisplatin administration (n = 6–8)**

**Treatment**	**NA**	**DOPAC**	**DA**	**5HIAA**	**HVA**	**5HT**
**Area postrema**						
Saline	0.610 ± 0.014	0.382 ± 0.111	0.590 ± 0.146	0.158 ± 0.036	0.913 ± 0.095	0.062 ± 0.034
Cisplatin	1.879 ± 1.622	0.312 ± 0.183	0.080 ± 0.030	0.316 ± 0.101^#^	0.555 ± 0.188	0.282 ± 0.120
MCP 30 mg	0.023 ± 0.005	0.017 ± 0.006	0.025 ± 0.012	0.005 ± 0.001*	0.121 ± 0.063*	0.023 ± 0.001
ZO-ActFr 50 mg	0.605 ± 0.221	0.080 ± 0.027	1.147 ± 0.615	0.013 ± 0.001***	0.171 ± 0.133	0.006 ± 0.002*
**Brain stem**						
Saline	0.094 ± 0.022	0.060 ± 0.020	0.175 ± 0.078	0.060 ± 0.021	0.060 ± 0.016	0.010 ± 0.003
Cisplatin	0.094 ± 0.024	0.173 ± 0.136	0.030 ± 0.001	0.036 ± 0.004^###^	0.028 ± 0.003	0.131 ± 0.020^###^
MCP 30 mg	0.119 ± 0.033	0.027 ± 0.006	0.044 ± 0.012	0.007 ± 0.001***	0.066 ± 0.031	0.019 ± 0.002
ZO-ActFr 50 mg	0.633 ± 0.050	0.015 ± 0.002	0.133 ± 0.077	0.018 ± 0.002***	0.147 ± 0.054	0.037 ± 0.004***
**Intestine**						
Saline	0.194 ± 0.059	0.067 ± 0.020	0.090 ± 0.064	0.076 ± 0.058	0.056 ± 0.025	0.049 ± 0.016
Cisplatin	0.222 ± 0.044	0.015 ± 0.003	0.022 ± 0.005	0.295 ± 0.024^###^	0.038 ± 0.004	0.665 ± 0.125^###^
MCP 30 mg	0.138 ± 0.039	0.054 ± 0.025	0.059 ± 0.018	0.097 ± 0.022	0.198 ± 0.102	0.062 ± 0.013
ZO-ActFr 50 mg	0.328 ± 0.036	0.088 ± 0.109	0.005 ± 0.055	0.030 ± 0.013***	0.124 ± 0.066	0.049 ± 0.021***

Values significantly different compared to cisplatin control are indicated as *p < 0.05, ***p < 0.001, while Values significantly different as compared to basal level are indicated as ^#^p < 0.05, ^###^p < 0.001 (ANOVA followed by Tukey post hoc analysis).

### Effect of standard MCP or ZO-ActFr on the level of neurotransmitters and their metabolites in the brain areas and small intestine at 18^th^ hour of cisplatin administration

Cisplatin increased the level of dopamine significantly (P < 0.001) in the AP, while a non-significant trend towards increase was observed in the areas of BS and intestine. 5HT concentrations were also raised in the brain area of BS (P < 0.05) and intestine (P < 0.001), without effecting the levels of NA, DOPAC, 5HIAA, HVA in the areas of BS and intestine and 5HT in the AP (Table 4). Treatment with standard metoclopramide (MCP; 30 mg/kg) significantly decreased the upsurge of DA at the brain area of AP (P < 0.001) and BS (P < 0.01). Furthermore a decrease in the concentration of 5HIAA and 5HT was also observed in the brain area of AP (P < 0.05 – 0.01) and intestine (P < 0.001) as compared to cisplatin control (Table 4). Pre-treatment with ZO-ActFr at (50 mg/kg) reduced the contents of DA in the brain area of AP (P < 0.001) and 5HT in the areas of BS and intestine (P < 0.001) as compared to cisplatin control (Table 4).

**Table 4:** **Effect of ZO-ActFr (50 mg/kg) or standard MCP (30 mg/kg) administered 30 mins before cisplatin challenge, on the level of neurotransmitters and their metabolites (ng/mg tissue wet weight) in the brain areas and intestine in pigeons at t = 18 hr of cisplatin administration (n = 6–8)**

**Treatment**	**NA**	**DOPAC**	**DA**	**5HIAA**	**HVA**	**5HT**
**Area postrema**						
Saline	0.610 ± 0.014	0.382 ± 0.111	0.590 ± 0.146	0.158 ± 0.036	0.913 ± 0.095	0.062 ± 0.034
Cisplatin	0.268 ± 0.073	0.026 ± 0.010	7.366 ± 1.500^###^	0.188 ± 0.053	0.556 ± 0.114	0.181 ± 0.052^##^
MCP 30 mg	0.023 ± 0.005	0.017 ± 0.006	0.025 ± 0.012	0.005 ± 0.001*	0.121 ± 0.063*	0.023 ± 0.001
ZO-ActFr 50 mg	1.304 ± 1.014	0.229 ± 0.081	0.846 ± 0.407***	0.082 ± 0.044	1.513 ± 0.817	0.127 ± 0.071
**Brain stem**						
Saline	0.094 ± 0.022	0.060 ± 0.020	0.175 ± 0.078	0.060 ± 0.021	0.060 ± 0.016	0.010 ± 0.003
Cisplatin	0.067 ± 0.004	0.001 ± 0.000	0.175 ± 0.026	0.034 ± 0.003	0.009 ± 0.001	0.121 ± 0.010^###^
MCP 30 mg	0.119 ± 0.033	0.027 ± 0.006	0.044 ± 0.012	0.007 ± 0.001***	0.066 ± 0.031	0.019 ± 0.002
ZO-ActFr 50 mg	0.014 ± 0.008	0.001 ± 0.000	0.080 ± 0.068	0.001 ± 0.000	0.009 ± 0.005	0.002 ± 0.000***
**Intestine**						
Saline	0.194 ± 0.059	0.067 ± 0.020	0.090 ± 0.064	0.076 ± 0.058	0.056 ± 0.025	0.049 ± 0.016
Cisplatin	0.223 ± 0.036	0.005 ± 0.001	0.151 ± 0.042	0.329 ± 0.054	0.060 ± 0.007	0.463 ± 0.098^###^
MCP 30 mg	0.138 ± 0.039	0.054 ± 0.025	0.059 ± 0.018	0.097 ± 0.022	0.198 ± 0.102	0.062 ± 0.013
ZO-ActFr 50 mg	0.172 ± 0.064	0.001 ± 0.000	0.485 ± 0.218	0.009 ± 0.006	0.050 ± 0.030	0.017 ± 0.011***

Values significantly different compared to cisplatin control are indicated as *p < 0.05, ***p < 0.001, while Values significantly different compared to basal level are indicated as ^##^p < 0.05, ^###^p < 0.001 (ANOVA followed by Tukey post hoc analysis).

## Discussion

In this study the extract of *Zingiber officinale* (*ZO*) was screened against cisplatin induced Retching plus Vomiting (R + V) in pigeons. *Zingiber officinale* (*ZO*; family *Zingiberaceae*) commonly known as ginger; the plant rhizome and is distributed and cultivated in Pakistan, India, Malaysia, China, Taiwan and Bangladesh. The standardization of ginger extract is reporting the quantities of gingerols in concentration ~ 60 mg/g of extract [36]. In our study, we used acetone fraction of *ZO* based on the study of Sharma and Co-workers [25], where acetone fraction is reported to be more effective to attenuate cisplatin induced emesis in dogs. Ginger have been screened in postoperative nausea and vomiting in clinics and is found to be superior to placebo and equally effective as metoclopramide [29]. In this study, the dose of 50 mg was found to be highly effective in attenuating cisplatin induced R + V, nonetheless longer but moderate protection i.e. upto 16 hr was observed with 25 mg dose. There are several lines of evidences explaining the anti-emetic effect of ginger; in animal models ginger is shown to enhance gastrointestinal transport i.e. having gastroprokinetic properties. Furthermore, ginger is having anti-hydroxytryptamine activity in the isolated ileal segments [37] as galanolactone, one of the component of ginger, has been proved to be a competitive antagonist at ileal 5HT_3_ receptors. Thus anti-emetic effects could be brought about by antagonism at 5HT_3_ receptors in the gastrointestinal tract [37,38]. Furthermore, ginger has also been found to be having inhibitory action on substance P and the expression of NK_1_ receptors [27]. Our results of current study are indicative of promising anti-emetic activity of ginger acetone extract against cisplatin induced vomiting in the pigeon. Ginger acetone extract was selected to find out its intrinsic antiemetic activity in line with its impact on central and peripheral neurotransmitters (and metabolites) involved in the act of vomiting, because the said fraction was found to be more effective to attenuate cisplatin induced vomiting in dogs as reported by Sharma and co-workers [25].

Metoclopramide (MCP), which is a clinically relevant anti-emetic with dopamine and 5-HT_3_ receptor antagonist properties [39] was used as a positive control. The dose of MCP that we selected is higher than required to antagonize cisplatin induced emesis in other species [40], and was based on a previous study in the pigeon showing activity against reserpine-induced emesis [2]. The metoclopramide was selected as standard because of the intrinsic emetic activity of 5HT_3_ receptor antagonists in pigeon (unpublished data).

As evident from the literature, neurochemical mediators of various types are responsible in vomiting circuits for CIV, especially serotonin (5HT), dopamine, substance P and prostaglandins are postulated to contribute in its genesis [6,26,41,42]. The emetogenic anti-cancer agent cisplatin induces biphasic vomiting both in human [43] and in other vomiting species [27,44-46]. Neural analysis of cisplatin control group in pigeon model indicated the upsurge of serotonin in the brain areas of BS and intestine at acute time point (03 hr) suggesting the neurotransmitter serotonin (5HT) as the triggering mediator for acute phase response in pigeons. The increase in 5HT concentration in our study is in line with the previous findings in animal models where 5HT_3_ receptor antagonists (ondansetron, granisetron, palonosetron etc.) are found to be effective against acute phase of CIV [10]. Furthermore, at delayed time point (18 hr) in pigeon the increase in the concentration of dopamine at the level of AP and serotonin in the brain area of BS and in the intestine is indicating the differential involvement of neurotransmitters at this time point; as CIV is thought to be a multifactorial phenomenon and both the phases of vomiting are mechanistically different [46], that’s why the prophylactic and intermittent administration of single antiemetic agent fails to provide complete control against cisplatin induced vomiting in clinics.

The pungent constituents collectively known as gingerols have been reported to be having inhibitory effects on the upsurge of substance P both centrally and peripherally and NK_1_ receptors expression in the vomit model of mink [27]. In our study, ZO-ActFr (50 mg) provided maximum protection against cisplatin induced R + V in pigeons, where the protection observed was ~ 58.13%. ZO-ActFr (25 mg) also resulted in the decrease in jerks (indicative of vomiting intensity) and reduced the weight loss significantly. The reduction in jerks and weight loss are the secondary parameters supporting the antiemetic profile of ginger acetone extract. Furthermore, the impact of ZO-ActFr on the major neurotransmitter mediators including dopamine and serotonin is encouraging and is in support for its anti-emetic effect where ZO-ActFr (50 mg) decreased the concentration of 5HT and 5HIAA in the brain areas (AP & BS) and intestine at acute time point (3 hr). Moreover, at delayed time point (18 hr) resulted in the decreased concentration of dopamine in the brain area of AP, while 5HT decrease was observed at the level of BS and in the intestine.

## Conclusions

In conclusion, *ZO* acetone fraction attenuated cisplatin induced vomiting in the pigeon which is mediated by both central and peripheral anti-serotonergic and anti-dopaminergic components in a blended manner at the two different time points. At the acute time point (3^rd^ hr), dominantly the anti-serotonergic effects were seen centrally in the area postrema and brain stem as well as peripherally at the level of intestine, while at the delayed time point (18^th^ hr) anti-serotonergic effects were observed centrally in the brain stem and peripherally in the intestine where centrally in the area postrema there was anti-dopaminergic aftermath.
